# Deciphering the maternal ancestral lineage of Greek Cypriots, Armenian Cypriots and Maronite Cypriots

**DOI:** 10.1371/journal.pone.0292790

**Published:** 2024-02-05

**Authors:** Irene Moutsouri, Panayiotis Manoli, Vasilis Christofi, Evy Bashiardes, Anna Keravnou, Stavroulla Xenophontos, Marios A. Cariolou

**Affiliations:** Department of Cardiovascular Genetics and The Laboratory of Forensic Genetics, The Cyprus Institute of Neurology and Genetics, Nicosia, Cyprus; Al Muthanna University, IRAQ

## Abstract

Cyprus was conquered from several populations because of its special geographical location. In this study, 406 unrelated Cypriot samples were tested based on their mitochondrial DNA. In more detail, 185 were Greek Cypriots, 114 Armenian Cypriots and 107 Maronite Cypriots. This is the first time where the mitochondrial DNA of Greek Cypriots, Armenian Cypriots and Maronite Cypriots is compared with the aim of characterizing the maternal ancestry of Cypriots. The control region of the mtDNA is the most informative in terms of studying maternal ancestry and consists of three hypervariable regions (HVS-I, HVS-II, HVS-III). The hypervariable regions can provide important information regarding the maternal ancestor of the tested samples. The entire control region of the mtDNA was used to determine the mitotypes and subsequently the haplogroups of all the Cypriot DNA samples. Based on the aforementioned analyses, Greek Cypriots were found to be genetically closer to Armenian Cypriots, while Greek Cypriots and Armenian Cypriots showed moderate genetic differentiation with Maronite Cypriots. The most prevalent haplogroups among Cypriots were haplogroups H and U, while R0 is common but in different frequencies for Greek Cypriots, Armenian Cypriots and Maronite Cypriots. It is proposed that the maternal ancestor may have originated during the Neolithic period and/or the Bronze age.

## Introduction

Cyprus is considered as one of the oldest and the third largest island in the Mediterranean region. Its special geographical location played a crucial role in bringing the island in close contact with the first centres of culture [1], and was the reason for it to be conquered by Assyrians, Crusaders, Turks and other populations.

According to archaeological excavations, Cyprus was inhabited, for the first time, during the Neolithic era in 8000 BC [1]. The mining of copper changed the history of the island, since neighbouring countries developed high interest in taking advantage of this metal. The Phoenicians, a seafaring and commercial population of the Eastern Mediterranean, arrived in Cyprus in 1050 BC as traders and to promote their economic and political interests. Subsequently, in 707–650 BC, Cyprus came under the rule of the Assyrians. Cyprus was freed from the Assyrians in 650 BC, but its freedom was lost to the Egyptians who conquered the island in 569 BC [2]. The Roman period began in 50 BC until 330 AD when Cyprus came under the Byzantine Empire in 330–1191. During the Byzantine era, the first Maronites and Armenians came to the island, in the 7^th^ and the 6^th^ century, respectively [3]. The first wave of Maronites arrived in Cyprus around 686 and then in 938, following religious clashes in Syria and Lebanon. In 1192 the Frankish rule began, when Richard the Lionheart sold Cyprus to the French nobleman Guy de Lusignan, until 1489. The last big wave of Maronites coming to Cyprus took place during the Frankish Era [4]. The traditional language of Maronite Cypriots is Cypriot Maronite Arabic (CMA). With respect to religion, they are in full communion with the Catholic church of Rome. The first wave of Armenians arrived in Cyprus in 578 from Arzanene Armenia. Armenians, also came to Cyprus from Western Europe, Cilicia and the Levant in 1192 [5]. Between the Byzantine and the Frankish Era more Armenians settled the island through several historical events. The Venetians directly ruled the island in 1489 but the island lived under the Ottoman Empire, since Ottomans had conquered all the countries of the East [1]. In 1570, the Turkish army arrived on the island and in 1571 Cyprus officially came under the Ottoman Empire until 1878 when Turkey transferred Cyprus under the authority of the United Kingdom. After the Armenian Genocide in 1915, a big wave of Armenians from Cilicia, Smyrna and Constantinople arrived on the island. During the periods 1947–1949 and 1956–1957, a new wave of Armenians arrived from Palestine and Egypt respectively [5]. Following the independence of Cyprus in 1960, two ethnic communities were established; the Greek Cypriot and the Turkish Cypriot communities. Maronite Cypriots, Armenian Cypriots and Latin Cypriots were recognised as religious groups who chose to belong to the Greek Cypriot community.

The latest documented reports show that the number of Maronite Cypriots living in Cyprus is 4,800 [6], Armenian Cypriots is 2,600 [6], Latin Cypriots is 900 [6] and Greek Cypriots 918,100 [7]. The Turkish Cypriots are estimated to be approximately 203,000. This study focused on the analysis of the mitochondrial DNA (mtDNA) of Greek Cypriots, Armenian Cypriots and Maronite Cypriots. Following the 1974 military invasion and occupation of Cyprus by Turkey, the majority of Turkish Cypriots live in the Turkish-occupied area of Cyprus rendering sample collection from Turkish Cypriots difficult. With respect to Latin Cypriots, due to the small number of individuals living in Cyprus, recruitment was more difficult, therefore, their genetic ancestry will be studied in the future. The data from a former study, with fewer samples regarding the mtDNA of Greek Cypriots was expanded through our current study [8]. To our knowledge, this is the first study that focused simultaneously on the mtDNA of Greek Cypriots, Armenian Cypriots and Maronite Cypriots. In the current study, we sought to perform a comprehensive analysis on the mtDNA of the examined Cypriot samples (Greek Cypriots, Armenian Cypriots, Maronite Cypriots), including genetic distances, haplogroup frequencies and hence, migration paths.

### Mitochondrial haplotypes—Mitotypes

The mutations identified in the three hypervariable regions comprise the mitotype of a given mtDNA sample. Similar mitotypes belong to the same haplogroup, thus, indicating that they share a common ancestor [9]. Mitochondrial DNA sequences determined today, can be traced back to a maternal mtDNA ancestor 120.000 years ago [10]. It has been determined that the current mtDNA sequences may carry approximately 40–70 mutations when compared to the ancestral human mtDNA sequence [11], which translates to about 1 mutation per 2500 years or 100 human generations [12].

Mitochondrial DNA haplogroups provide information regarding the common ancestor, since the mutations or polymorphisms comprising the mitotypes are inherited without recombination from the mother to all of her children [13]. The nomenclature of mtDNA haplogroups was introduced in the 1990s with letters A-G assigned to variations observed in Asian and American lineages [14], letters H-K were assigned to Europe [15] whereas only a single letter, L, was assigned to describe the highest level of variation observed in Africa [16].

## Materials and methods

### Ethics approval, sample collection, DNA extraction and quantitation

The study was approved by the Cyprus National Bioethics Committee (CNBC) with proposal number: EEBK/EΠ/2015/06. Informed written consent was obtained from all participants who provided DNA samples. Inclusion criteria were as follows: unrelated individuals, who were 18 years old or above, born in Cyprus, with a Greek Cypriot, Armenian Cypriot or Maronite Cypriot maternal ancestry. The design and execution of this project fulfilled all the personal data protection requirements stipulated by the General Data Protection Regulation (GDPR 2016/679). A total of 406 unrelated Cypriot samples were collected by organizing several field trips. From the 406 samples, 185 were Greek Cypriots, 114 Armenian Cypriots and 107 Maronite Cypriots DNA extraction was carried out using a QIAGEN BioRobot Universal (BRU) with QIAamp 96 DNA Swab BioRobot Kit or by using QIAamp DNA investigator kit from QIAGEN [17, 18]. The extracted total nuclear DNA was quantified using an internally validated published method [19], and ran on the AB-7500 RT PCR System.

### Mitochondrial DNA genotyping and EMPOP submission

The entire control region (CR) of the human mtDNA,16024–576 bp, was amplified and sequenced according to the published revised and extended guidelines for mtDNA typing [20]. One set of primers was used for the entire control region amplification, F15989 and R599 or R670 (S1 Table), while three sets of primers were used for the sequencing of each hypervariable region, HVS-I, HVS-II, and HVS-III (S1 Table). The PCR reaction was carried out in a final volume of 25μl. PCR was performed on the Applied Biosystems Veriti thermal cycler. Appropriate numbers of reagent blanks and negative controls were used to detect any contamination in the reagents used and therefore, the validity of the data generated. The amplified PCR products were visualized using 2% agarose gels to assess successful amplification.

The amplified PCR products were purified using the QIAGEN QIAquick PCR purification kit (Qiagen). The purified PCR products were then sequenced using one set of forward (F) and reverse (R) primers in separate reactions (as shown in S1 Table) and the ABI BigDye Terminator Cycle Sequencing Kit. The products were then purified from unincorporated dyes using Centriflex Columns (Edge Biosystems) and then electrophoresed on the AB 3130xl Genetic Analyzer (Applied Biosystems). The mtDNA sequences were aligned and compared to the revised Cambridge Reference Sequence (rCRS), using the software “SeqScape”. The nomenclature guidelines for mtDNA typing were used as described by Parson et al. 2014 [20].

The mitotypes of all the 406 analyzed Cypriot samples were submitted to the EMPOP-mtDNA database (https://empop.online/). The EMPOP database is the largest database regarding searchable mtDNA mitotypes from all over the world that have passed a quality control inspection. The accession numbers of the three Cypriot datasets are EMP00873 (Greek Cypriots), EMP00872 (Armenian Cypriots) and EMP00874 (Maronite Cypriots) [20–22].

A previous study investigated the mtDNA of Greek Cypriots and Greeks [8]. In this previous study, the mtDNA HVI-HVIII of 91 Greek Cypriots and 319 Greeks was sequenced. Since the mtDNA mitotype frequencies of Greek Cypriots between the current study and the previous study were similar, these two datasets were pooled together in a combined new dataset that included a total of 276 Greek Cypriot mtDNA sequences. Following this, all the calculations were performed using the combined, enriched, dataset of 276 samples. The same approach was used for the Greek mtDNA samples where datasets from two studies were pooled together into a new combined dataset of 417 mtDNA sequences from Greek individuals [8, 23].

### Haplogroup assignment and analysis

The haplogroup for each Cypriot mtDNA sample was determined after the sequencing data were submitted to EMPOP. Haplogroup frequencies were calculated for the Greek Cypriots, Armenian Cypriots and Maronite Cypriots. Mitochondrial DNA haplogroup frequencies were also obtained from publications providing datasets from Armenia (including both ancient and modern samples) [24] and countries of the Mediterranean region. In this study, we chose to focus on countries around Cyprus that are also washed by the Mediterranean Sea. Armenia was exempted due to the close historical ties of Armenian Cypriots with this Country. Hence forth, Armenia and countries of the Mediterranean region (Albania [25], Algeria [26], Bosnia and Herzegovina [27], Croatia [28], Cyprus [8], Egypt [29], France [30], Greece [8, 23], Italy [31], Lebanon [32], Libya [33], Montenegro [34], Morocco [33], Palestine [33], Slovenia [35], Spain [36], Syria [37], Tunisia [38], Turkey [39]) will be designated in the current paper as countries of interest. The haplogroups from the countries of interest were compared with those from Cyprus. By using the haplogroup frequencies, mappies were generated using R programming and R studio [40–42].

### Genetic distances

The mitotypes of all Cypriot samples were obtained using all hypervariable segments (HVS-I, HVS-II and HVS-III). For comparative purposes, mitotypes from the countries of interest were obtained from previous publications [8, 23–27, 29–39, 43, 44]. Since most of the populations had available mitotypes only for the HVS-I, we have used data only for the HVS-I to calculate the genetic distances, using the Arlequin software [45–47]. There are three levels of differentiation when considering genetic distances. Low genetic differentiation is found in populations with genetic distances between 0–0.05. Values in the range of 0.05–0.15 show moderate genetic differentiation, while values greater than 0.15 indicate high levels of genetic differentiation [48]. The statistical significance level of p-values was defined using 10.000 permutation tests in the software options. For a better representation of the genetic distances of all populations, a heatmap was created using R programming and R studio [40–42]. A colour scale was added to distinguish the genetic correlation between the populations. The genetic correlation between Cypriots (Greek Cypriots, Armenian Cypriots, Maronite Cypriots) was analyzed using the principal component statistical function in R. The statistical results obtained were presented on a 3D PCA plot. The data used in this analysis were all the SNPs identified by sequencing HVS-I, HVS-II and HVS-III of the Cypriot samples. Both analyses, principal component statistical function and plot, were performed using R programming and R studio [40–42]. Mitochondrial DNA haplogroup paths were created using R programming and R studio [40–42]. The paths include all the mitochondrial haplogroups found in all Cypriot samples.

## Results

### Intra population analysis

This study aimed to look at the mtDNA of 276 Greek Cypriots, 114 Armenian Cypriots and 107 Maronite Cypriots. The genetic distances, representing the genetic differentiation between these mtDNA samples are shown in Table 1.

**Table 1:** Calculated genetic distances among datasets of Cypriots.

Dataset #1	Dataset #2	Fst value
Greek Cypriots [n = 276]	Armenian Cypriots [n = 114]	0.023
Greek Cypriots [n = 276]	Maronite Cypriots [n = 107]	0.068
Armenian Cypriots [n = 114]	Maronite Cypriots [n = 107]	0.076

**Fig 1:**
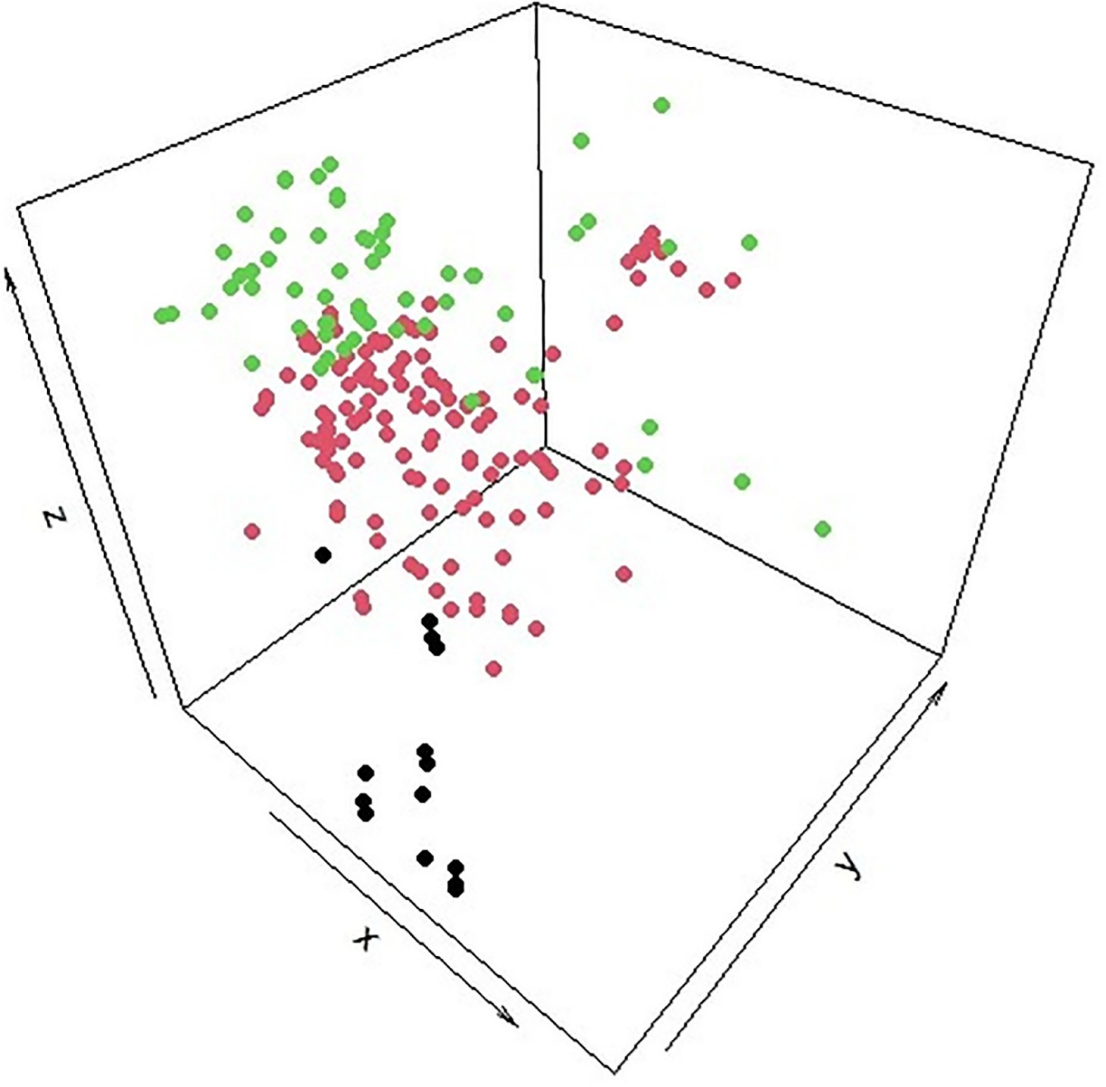
Principal Component analysis using SNP data. The genetic correlation between Greek Cypriots, Armenian Cypriots and Maronite Cypriots is shown. The green colour represents the Armenian Cypriots, the red colour the Greek Cypriots and the black colour the Maronite Cypriots.

The genetic distance between Maronite Cypriots and Greek Cypriots showed moderate genetic differentiation, as well as the genetic distance between Maronite Cypriots and Armenian Cypriots. In contrast, Armenian Cypriots and Greek Cypriots showed low genetic differentiation with a value in the range of 0–0.05. This indicated that Greek Cypriots are genetically closer to Armenian Cypriots compared to Maronite Cypriots. The low genetic differentiation between Greek Cypriots and Armenian Cypriots was also shown in the PCA plot, in Fig 1. In contrast, Greek Cypriots and Maronite Cypriots as well as Armenian Cypriots and Maronite Cypriots showed moderate genetic differentiation. As indicated above and also shown in Fig 1, all Cypriot samples have a genetic relationship but the correlation between them differs. Finally, based on these results, Greek Cypriots and Armenian Cypriots have more mitotypes in common, compared with Maronite Cypriots.

### Inter population analysis

Additional genetic distances were calculated between Cypriots and the countries of interest [8, 23–27, 29–39, 43, 44]. The calculated genetic distances are shown in Table 2. The entire extensive analysis using the Arlequin program for all mtDNA datasets from the countries of interest are provided in S2 Table.

**Table 2:** Calculated genetic distances between Cypriots and the countries of interest.

Dataset	Greek Cypriots [n = 276]	Armenian Cypriots [n = 114]	Maronite Cypriots [n = 107]
Greek Cypriots [n = 276]	0.000	0.023	0.068
Armenian Cypriots [n = 114]	0.023	0.000	0.076
Maronite Cypriots [n = 107]	0.068	0.076	0.000
Albania [n = 148]	0.021	0.027	0.071
Algeria [n = 240]	0.025	0.031	0.071
Armenia (ancient samples) [n = 50]	0.013	0.020	0.061
Armenia (modern samples) [n = 61]	0.021	0.028	0.071
Bosnia [n = 239]	0.013	0.020	0.060
Croatia [n = 488]	0.013	0.020	0.062
Egypt [n = 277]	0.012	0.019	0.061
France [n = 210]	0.019	0.025	0.067
Greece [n = 417]	0.015	0.021	0.063
Herzegovina [n = 130]	0.018	0.025	0.071
Italy [n = 395]	0.021	0.028	0.069
Lebanon [n = 195]	0.015	0.021	0.062
Libya [n = 83]	0.061	0.069	0.112
Montenegro [n = 139]	0.017	0.023	0.063
Morocco [n = 149]	0.047	0.054	0.059
Palestine [n = 110]	0.015	0.022	0.062
Slovenia [n = 402]	0.021	0.027	0.068
Spain [n = 403]	0.028	0.034	0.067
Syria [n = 234]	0.011	0.018	0.057
Tunisia [= 58]	0.039	0.046	0.084
Turkey [n = 224]	0.013	0.019	0.059

As shown in Table 2, Maronite Cypriots showed moderate genetic differentiation with all of the 20 countries of interest, compared to Armenian Cypriots that showed low genetic differentiation with all countries except from Libya and Morocco. Greek Cypriots showed low genetic differentiation with all countries except from Libya. Maronite Cypriots originated from Lebanon and Syria [4]. The genetic distances between Maronite Cypriots and Lebanon and Syria fall within the range of 0.05–0.15, supporting a moderate genetic differentiation.

Armenian Cypriots showed low genetic differentiation with various countries such as Egypt, Turkey, Syria and, as expected, with Armenian populations. Samples from Armenia (ancient and modern samples) [24] were included in the calculations. The modern samples were obtained from the Ararat region of Armenia. The calculated genetic distance is 0.020 between Armenian Cypriots and Armenia (ancient samples), while the genetic distance between Armenian Cypriots and Armenia (modern samples) was 0.028. Both values indicate low genetic differentiation. Genetic distances between Greek Cypriots and Greece, Turkey, Syria, Lebanon, Egypt, Armenia (ancient samples), Croatia and Bosnia showed low genetic differentiation with values between the range of 0.010–0.015.

**Fig 2:**
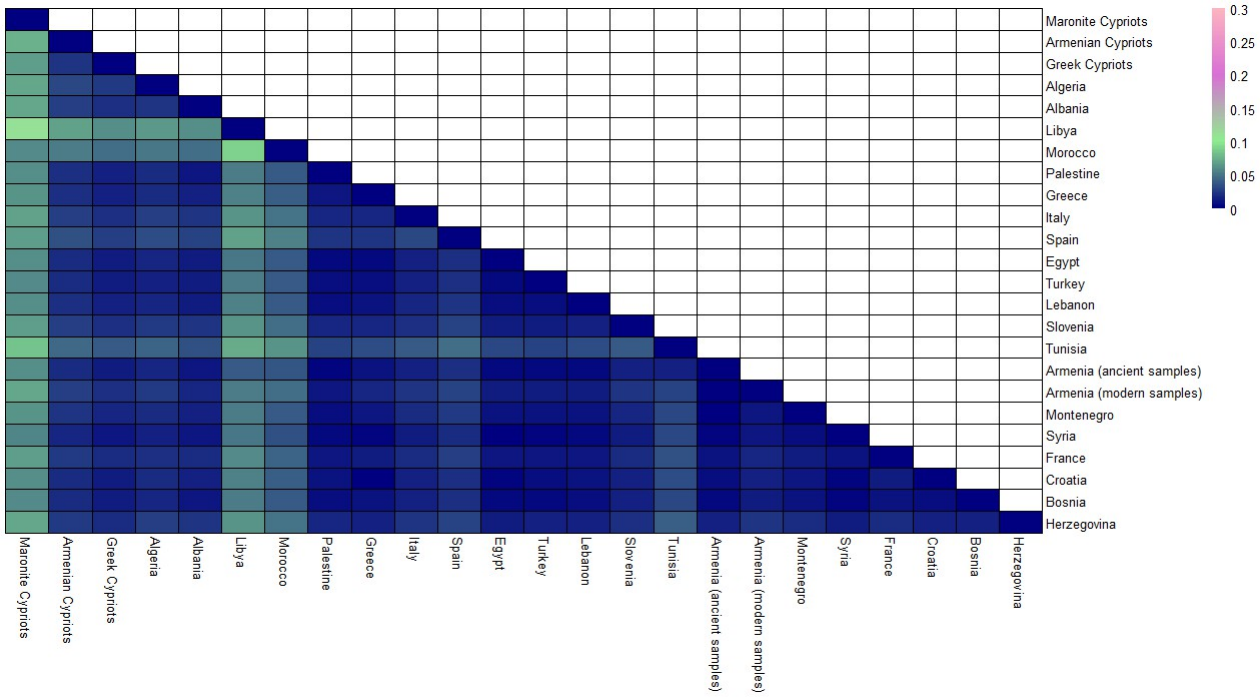
Heatmap using genetic distances. The genetic differentiation is shown using the calculated genetic distances between Cypriots and the countries of interest. The colour scale indicates the three level of genetic differentiation.

Since there is a range of values in the genetic distances among Cypriots and the countries of interest, a heatmap was created, shown in Fig 2, to represent the values for each population. A colour scale was added to determine the genetic correlation between colour and value. The dark blue represents the identical populations with values 0. The light green belongs to the population with low genetic differentiation. Genetic distances between the range 0–0.05 are shown in the colour scale between the colours dark blue and light green. None of the comparisons showed great genetic differentiation with genetic distance values above 0.15, thus none of the other colours in the scale is shown in Fig 2. All genetic distance values were between the range of 0–0.11, showing low and moderate genetic differentiation.

### Mitochondrial DNA haplogroups

Greek Cypriots, Armenian Cypriots and Maronite Cypriots were assigned collectively to 129 total haplogroups and subclades, thus frequencies were calculated for each Cypriot group.

**Fig 3:**
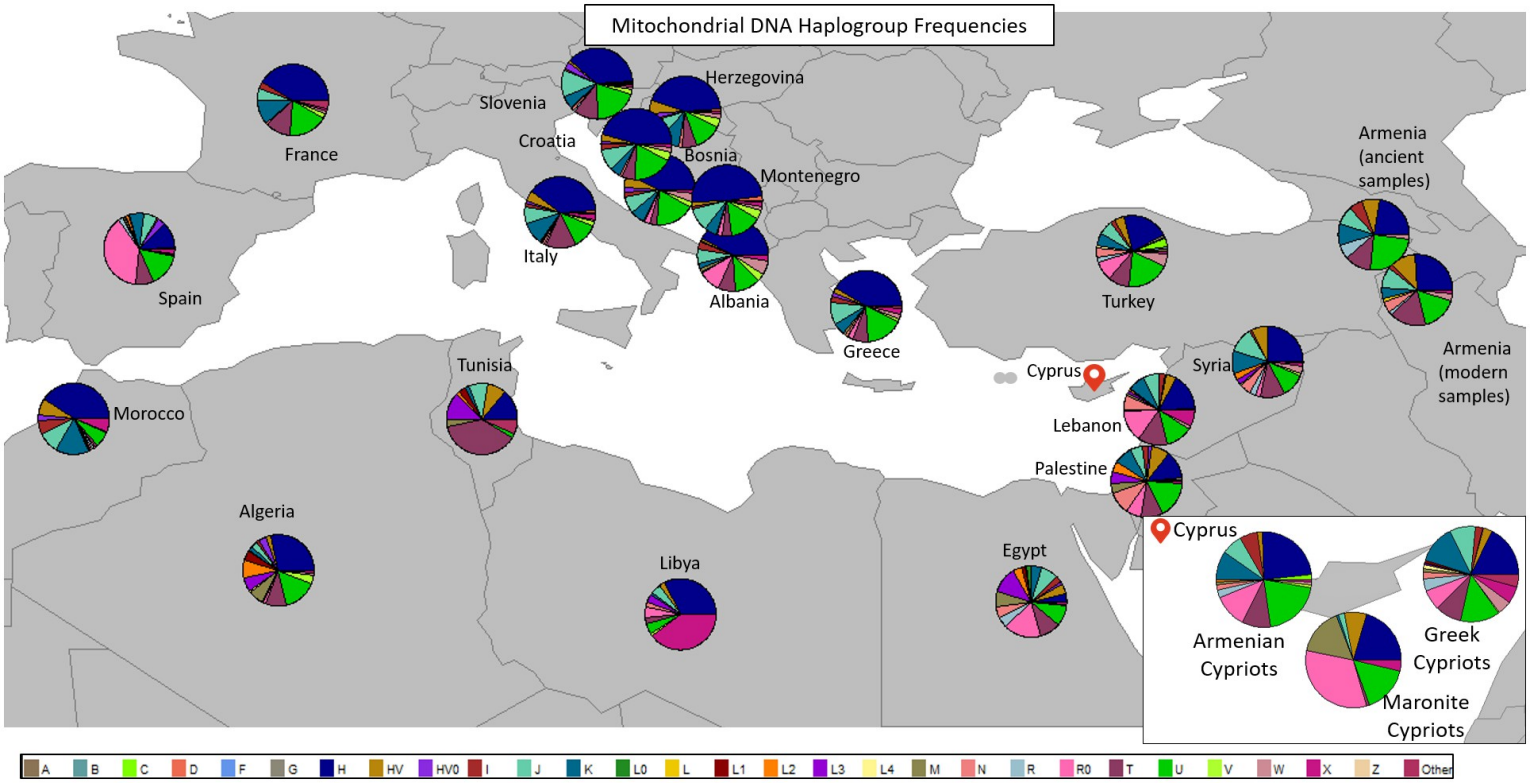
Mitochondrial DNA haplogroup frequencies among Cypriots and countries of interest. The mappies show the haplogroup frequencies. The zoomed in portion shows the haplogroup frequencies of the tested Cypriot samples. Each haplogroup is indicated with different colour.

All haplogroup frequencies are shown in Fig 3 (S3 Table) representing the percentage of each mitochondrial haplogroup for each country. Furthermore, haplogroup frequencies were calculated between Cypriots and the countries of interest. The positioning of the mappies on the world map, in Fig 3, are not directly related to the geographical location from where the data were collected from.

The mitochondrial haplogroups of Cypriots are shown more clearly in the zoomed portion of Fig 3. In this Fig 3, the major haplogroups H, R0 and U, are presented with a high frequency in all tested Cypriot samples. In S4 Table, mitochondrial haplogroup subclades are shown in more detail, in order to see how the frequency of the major haplogroups diverged in the several haplogroup subclades. Haplogroup subclades of Greece, Lebanon, Syria and Turkey are also shown, which are the four closest countries to Cyprus.

Haplogroup H, is found with a frequency of 17.75% in Greek Cypriots, 23.68% in Armenian Cypriots and 20.56% in Maronite Cypriots (S3 Table). In Fig 3, the blue colour in the mappies represents haplogroup H which is dominant in all Cypriots. As shown, haplogroup H, was observed in almost all countries with different frequencies. The haplogroup H diverged in to a number of haplogroup subclades that vary at a frequency in accordance with a given sub-population. Armenian Cypriots diverged from the H haplogroup to the following subclades: H14a, H15,H1bd, H20a, H20a1a, H5a1e, H5n and H7c1. Greek Cypriots, from the H haplogroup diverged to subclades: H13a2c1, H14a, H15, h1bd, H1c, H20a1a, H27, H28, H2a2a1, H4d, H5, H5a1e, H5u, H6, H7c1 and H9 (S4 Table). Maronite Cypriots, diverged in only three H haplogroup subclades: H5u, H7c1 and H8 (S4 Table). Haplogroup R0, was observed with a frequency of 11.4% in Armenian Cypriots, 6.88% in Greek Cypriots and 32.71% in Maronite Cypriots (S3 Table). Haplogroup R0 diverged only in one subclade: R0a1a. In mappies, R0 is represented by light pink colour and was shown to be more frequent in Maronite Cypriots, followed by Armenian Cypriots and then by Greek Cypriots. The third mitochondrial haplogroup which is common in all tested Cypriot samples is haplogroup U. Haplogroup U, is found with a frequency of 20.18% in Armenian Cypriots, 13.41% in Greek Cypriots and 15.89% in Maronite Cypriots (S3 Table). This haplogroup diverged in several haplogroup subclades. Armenian Cypriots diverged in haplogroup subclades: U1, U1a, U1a1a, U1a1d, U1b, U2d, U3b1a, U3b2a1a, U3b3, U5b3 and U7a2a. Haplogroup U in Greek Cypriots diverged in the following haplogroup subclades: U1, U1b, U1b3, U3, U3c, U4, U4c2a, U5, U5a1, U5a2, U5b, U6, U7, U8b1a, U8b1a1 and U8b1b. In turn, Maronite Cypriots diverged in haplogroup subclades: U1b3, U3, U8b1a1 and U8b1a2b (S4 Table).

Haplogroup J is common between Greek Cypriots and Armenian Cypriots with frequencies of 7.02% and 8.70%, respectively (S3 Table), while in Maronite Cypriots this haplogroup was found with a lower frequency (1.87%). This is also shown in Fig 3 where in Armenian Cypriots and Greek Cypriots, haplogroup J, is dominant while in Maronite Cypriots it is shown to be present with a very low frequency.

Haplogroup subclade K1a is found with a high frequency in Greek Cypriots (11.96%) and Armenian Cypriots (7.89%), in contrast with Maronite Cypriots, where it was observed with a frequency lower than 1% (S4 Table). This also applies for haplogroup T, where in Armenian Cypriots and Greek Cypriots it is observed with high frequencies (9.65% and 13.04%, respectively) whereas in Maronite Cypriots it is observed with a low frequency (<1%). Maronite Cypriots are assigned to haplogroup subclade M1 with a frequency of 14.95%, while Armenian Cypriots and Greek Cypriots are assigned to this haplogroup with frequencies lower than 1%. Haplogroup I, is observed with a high frequency in Armenian Cypriots but in Greek Cypriots it is observed with a low frequency, 1.09%. Haplogroup I was not observed in Maronite Cypriots. Haplogroup HV is found with a high frequency in Maronite Cypriots, but in Armenian Cypriots and Greek Cypriots it was observed with a low frequency (~1–3%).

Greece, Lebanon, Syria and Turkey are the four countries which were selected to be shown in S4 Table with the detailed mitochondrial haplogroup subclades. The criterion for this selection was their close geographical position with Cyprus.

### Mitochondrial haplogroup paths

**Fig 4:**
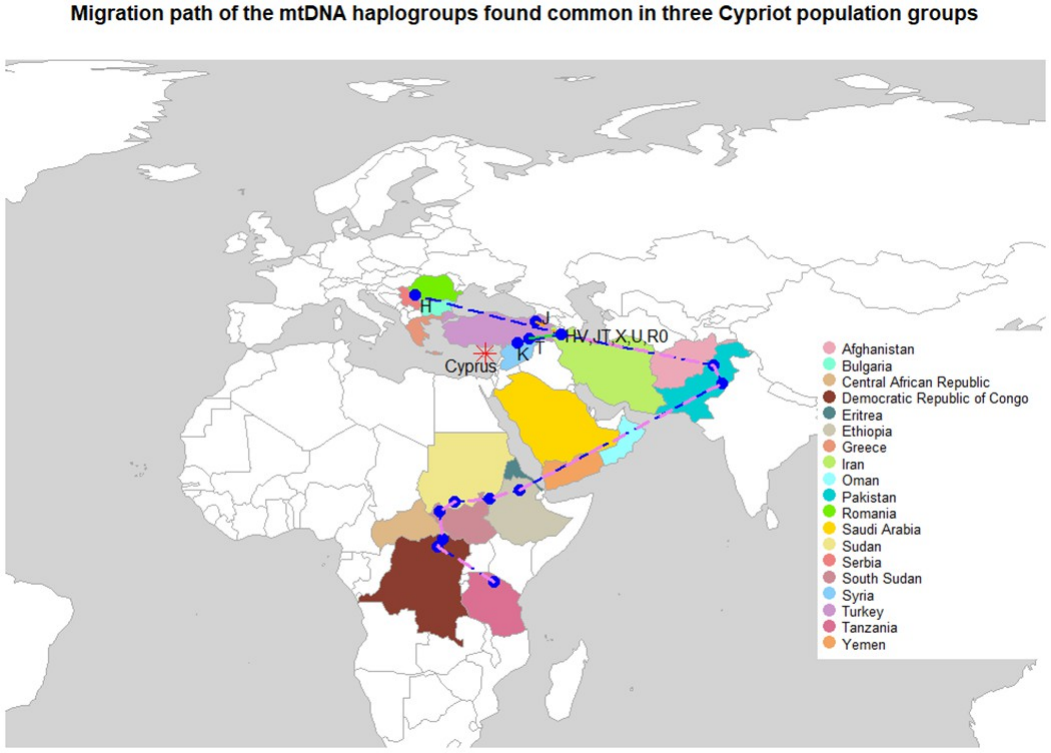
Migration path of the mitochondrial DNA haplogroups found common in Greek Cypriots, Armenian Cypriots and Maronite Cypriots. All mitochondrial haplogroups were generated from the African macro-haplogroup L. Haplogroups J, J K and T were branched from the haplogroups HV, JT, X, U, R0.

The major mitochondrial haplogroups observed in Greek Cypriots, Armenian Cypriots and Maronite Cypriots are haplogroups C, H, HV, I, J,K L1, L2, L3, L4, M, N, R, R0, T, U, V, W and X. The migration path of these mitochondrial haplogroups is shown in Fig 4. The first reported mitochondrial haplogroup was haplogroup L in Africa that diverged to haplogroups L1, L2, L3, L4, L5, L6 and L7 [49].

## Discussion

### Genetic distances between Cyprus and the countries of interest

The mitotypes of Greek Cypriots, Armenian Cypriots and Maronite Cypriots were compared with the countries of interest (as defined in this study) since Cypriots have a long history of several migrations. Maronite Cypriots originated mainly from Lebanon and Syria, however the genetic distances between these two countries were 0.062 and 0.057, respectively, indicating a moderate genetic differentiation between them. Similarly, the genetic distances between Maronite Cypriots and the countries of interest showed moderate genetic differentiation with values in the range of 0.05–0.15. Armenian Cypriots originated mainly from Armenia and various regions of Turkey. The calculated genetic distances showed that Armenian Cypriots were genetically close to the Turkish population with a genetic distance of 0.019. Genetic distances were calculated with two different population datasets, one from ancient samples of Armenia and one from modern samples of Armenia. These two population datasets between them showed low genetic differentiation with genetic distances in the range of 0–0.05. Low genetic differentiation was observed also among Armenian Cypriots and datasets from Algeria, Albania, Palestine, Greece, Italy, Spain, Egypt, Lebanon, Slovenia, Tunisia, Montenegro, Syria, France, Croatia, Bosnia and Herzegovina. Moderate genetic differentiation was shown between Armenian Cypriots and datasets from Libya and Morocco. Greek Cypriots showed low genetic differentiation with all countries of interest, except with the population of Libya, where a moderate genetic differentiation with a value of 0.061 was observed.

### Mitochondrial haplogroup analysis among Cypriots and the countries of interest

The three most prevalent mitochondrial haplogroups in Cypriots were haplogroups H, R0 and U, however, the observed frequencies of these haplogroups in each Cypriot dataset differs between them. These haplogroups were compared with the haplogroups of the four closest countries to Cyprus. Haplogroup H was found with a high frequency, between 16–40%, in Greece, Lebanon, Syria and Turkey. The subclade H14a, that was common in Greece, Lebanon and Syria, was also found in Armenian Cypriots and Greek Cypriots. The haplogroup subclade H20a was observed in the Armenian Cypriots, subclades H5, H6 and H9 were observed in Greek Cypriots and subclade H8 was observed in Maronite Cypriots. Subclade R0a1a was found in all Cypriots. Haplogroup U was observed in all of the four aforementioned countries with frequencies between 12–20%. Haplogroup subclades U1 and U1b were observed in both Armenian Cypriots and Greek Cypriots. The subclades U1b3 and U3 were found in Greek Cypriots and Maronite Cypriots. Haplogroup subclade U1a was common in Greece, Lebanon and Turkey and was found also in Armenian Cypriots. More specifically, in Armenian Cypriots the haplogroup subclades observed were U1a, U1a1a, U3b3 and U5b3 whereas in Greek Cypriots the subclades observed were U4, U5, U5a1, U5a1,U5a2, U5b, U6 and U7. Armenian Cypriots and Greek Cypriots showed low genetic differentiation with Greece, Lebanon, Syria and Turkey in contrast with Maronite Cypriots who showed moderate genetic differentiation.

### Mitochondrial haplogroup analysis between the countries of interest

The matrilineal most recent common ancestor for all living humans, Mitochondrial Eve was rooted in Africa approximately 150000 Years Before Present (YBP). This root was the large continent specific lineage macro-haplogroup L and diverged into four lineages: L0, L1, L2 and L3 which were specific for sub-Saharan Africa and were generated 100000 YBP. Two macro-haplogroups, M and N, were generated from the African haplogroup L3 approximately 65000 YBP [50]. Haplogroup N was directed to Eurasia, Asia and America, while haplogroup M was directed to Asia. Comparison of all tested Cypriot samples, indicated that haplogroup M was found at a high frequency in Maronite Cypriots and Algeria Haplogroup R originated from haplogroup N in Europe 60000 YBP that subsequently diverged into haplogroups including R0(HV, H,V), U(K), JT (J, T), H and V [51–54].

The majority of the European populations, belong to the major mtDNA haplogroups HV, U and JT [55, 56]. As shown from previous studies haplogroup H was found in the Middle East approximately 30–25000 YBP and was expanded to Europe 22000 YBP from the Near East, together with the second Palaeolithic wave [57]. Approximately 45% of the European populations, 20% of Turkey and Caucasus and 10% of the Gulf countries belong to haplogroup H [58]. As described by Achilli et al. [53], haplogroup H was also presented in the Neolithic admixture of agriculture from the Near East, the expansion of the Kurgan culture from southern Ukraine and the recent events of gene flow to northern India. In the present study, haplogroup H was found, with a high frequency in Greek Cypriots, Armenian Cypriots and Maronite Cypriots.

Haplogroup HV is more common in the Near East, Caucasus, South and East Europe. Haplogroup HV expanded during the Last Glacial Maximum (LGM) [59]. Haplogroup HV is also found in all Cypriots but the highest frequency was observed in Maronite Cypriots. Haplogroup HV, is frequent in Armenia (both ancient and modern samples), Bosnia, Herzegovina, Morocco, Palestine, Syria and Tunisia. Maronite Cypriots showed moderate genetic differentiation with these countries; therefore, they share a number of common mitotypes between them. Armenia, Palestine and Syria belong in the Near East region where haplogroup HV was common. A number of Maronite Cypriots originated from Syria, therefore, this may explain the high frequency of HV in Maronite Cypriots.

The subclade H1 (Haplogroup H), found with a high frequency in Scandinavian and the Southern Iberian Peninsula, was observed in Armenian Cypriots, Greek Cypriots and the Greek population. Subclades H2 and H6 of haplogroup H, are more frequent in the Caucasus and Eastern Europe. Haplogroup subclade H6 was observed in Greek Cypriots.

Haplogroup R0 is a major haplogroup of the macrohaplogroup R that branches into the major haplogroups HV, H and V. Haplogroup R0 was observed in the Near East, Caucasus and Central Asia with frequencies between the range of 10–30% [60]. Haplogroup R0 was observed with a high frequency in Armenian Cypriots, Maronite Cypriots and Greek Cypriots. Maronite Cypriots had the highest frequency among Cypriots assigned to haplogroup R0. This, most probably, reflects the high frequency of R0 in Lebanon and Syria. Maronite Cypriots originated mainly from these two countries.

A high percentage (40%) of the European populations belongs to the macrohaplogroups JT and U. Haplogroup J, of the macrohaplogroup JT, together with haplogroup V, were found at a low frequency in the European populations [54]. Based on previous studies [8, 23–25, 27, 29, 31–39, 43], haplogroup J was found at a high frequency in Armenian Cypriots, Greek Cypriots, Albania, Armenia (ancient samples), Armenia (modern samples), Bosnia, Herzegovina, Croatia, Egypt, Greece, Italy, Lebanon, Montenegro, Morocco, Slovenia, Syria, Tunisia and Turkey. The high frequency of haplogroup J supports the suggestion that the origin of Armenian Cypriots may well be Armenia and Turkey, where, in these two countries, haplogroup J is found with a high frequency.

Haplogroup U is the second most common haplogroup in Europe and it was reported with a high frequency in pre-agriculture Europe [61, 62]. The subclades of haplogroup U, were found at a low frequency in Early Neolithic farmers and in those from Central Europe [63] but with the resurgence of hunter-gatherers, the frequency of haplogroup U was increased during the Middle Neolithic period [62, 64]. Haplogroup U was found with a high frequency in all Cypriot samples. The observed high frequency of haplogroup U in Cypriots was expected since this haplogroup was observed in all the countries from which Armenian Cypriots and Maronite Cypriots originated from (Armenia, Egypt, Israel, Palestine, Syria and Turkey) and those populations (Egypt, France, Italy) that came to Cyprus through different historical events during the arrival of Phoenicians, Assyrians, Egyptians, Franks, Venetians and Ottomans. Haplogroup U, branches into subclades U1, U5, U6 and a subclade that branches into haplogroup clades U2, U3, U4’9, U7 and U8 [44]. U8 branches to haplogroup K [62]. The prevalence of the U subclades differs between populations. Haplogroup subclades U1 and U3 are prevalent in the Near East and based on previous studies [8, 23, 32, 39] are found in Greek, Lebanese and the Turkish populations. Subclades U1 and U3 were found in all Cypriot samples. The haplogroup subclades U4 and U5 are common in Europe. Ancient European hunter-gatherers were assigned to subclades U4 and U5 of haplogroup U [61, 62]. These two subclades are observed in Greeks, Lebanese and in Greek Cypriots. Subclade U5, among the Cypriots, was observed in Armenian Cypriots only. Subclade U5 is also found in the Turkish population. The presence of subclade U5 in Turks and Armenian Cypriots explains the low genetic distance between these two sample groups. The introduction of lineages including U2, U4, U5aT1 and H in Europe, occurred during the Late Neolithic and Bronze Age [64]. The subclade U2 is frequent in South Asia but the subclades U2d and U2e are found only in the Near East and Europe. The subclade U2d is observed in Armenian Cypriots, Lebanese and Turks. The mitochondrial Neolithic package composed from haplogroup subclades including K, J, HV and V were associated with the Early Neolithic period due to the transition to farming [65]. Haplogroup K is prevalent in Armenian Cypriots, Greek Cypriots, Armenia (ancient samples), Bosnia, Herzegovina, France, Italy, Lebanon, Morocco, Palestine, Slovenia, Spain and Syria. Haplogroup T is found at a high frequency in Armenian Cypriots and Greek Cypriots but not in Maronite Cypriots. Haplogroup T is also found with a high frequency in all countries of interest except Bosnia, Libya, Montenegro and Morocco.

### Shared ancestry among Cypriots

The most prevalent haplogroups among all Cypriots were haplogroups H and U. With lower prevalence, haplogroup R0 was also common among the tested Cypriot samples but the frequencies between them differed. Haplogroup H, that was observed in the Middle East, arrived in Europe 22000 YBP. People from Armenia, Egypt, Lebanon, Palestine, Syria and Turkey passed through Cyprus either as conquerors or through waves when Maronites and Armenians arrived to the island to settle. Haplogroup H was frequent in these countries, except Egypt. Armenian Cypriots and Greek Cypriots showed a close genetic correlation (< 0.05) with these populations while Maronite Cypriots were shown to have a moderate genetic differentiation (0.05–0.15). It is supported that haplogroup H is a Neolithic admixture from the Near East. Haplogroup U was found at a high frequency in pre-agriculture Europe and during the Middle Neolithic period approximately 7800–7300 YBP. Haplogroup U is frequent in countries from where Maronite and Armenians originated and in populations which came to Cyprus through different historical events. Subclades of haplogroup U that are found in all tested Cypriot samples are also prevalent in the Near East. Lineage U2, U4, U5 and haplogroup H were reintroduced in Europe in the Late Neolithic period and Bronze age. The subclades U4 and U5 are found in both Armenian Cypriots and Greek Cypriots, whereas U2 is found only in Armenian Cypriots. Haplogroup H was found in Greek Cypriots, Armenian Cypriots and Maronite Cypriots.

The tested Cypriot samples have a number of haplogroups in common and they showed genetic differentiation with different genetic distances. According to the mtDNA analysis, Greek Cypriots and Armenian Cypriots showed low genetic differentiation, in contrast with the genetic correlation between the Greek Cypriots and Maronites Cypriots and the Armenian Cypriots and Maronite Cypriots. Greek Cypriots and Armenian Cypriots compared with Maronite Cypriots, showed moderate genetic differentiation. This genetic relationship between the three Cypriot datasets was shown also in the PCA plot, that was performed using all the identified SNP data in the Cypriot samples tested. The Greek Cypriots and Armenian Cypriots resemble each other and the Maronite Cypriots are more different. The difference between Maronite Cypriots and Greek Cypriots and Armenian Cypriots may be due to the fact that Maronite Cypriots are more homogeneous (as documented by information provided on the consent forms) and tend to marry within their own religious group. The same correlation between Cypriots was also observed on the Y chromosome analysis. Greek Cypriots are more genetically closer based on the Y chromosome analysis with low genetic differentiation, while Greek Cypriots and Armenian Cypriots compared with Maronite Cypriots, showed moderate genetic differentiation.

Based on historical documentation, Cyprus went through major colonization events during the Neolithic period and Bronze Age [1]. This documentation affirms the expansion of particular mitochondrial DNA haplogroups (Haplogroups H and U) [53, 59, 62, 65] found with high frequency in the Cypriot population.

The most prevalent mitochondrial haplogroups among all Cypriots were haplogroups H and U. Based on these observations, it is proposed that the maternal ancestor of Cypriots, may have originated during the Neolithic period and/or Bronze age.

## Supporting information

S1 Table Primer sequences which were used for Sanger Sequencing. A combination of a forward and reverse primers from the table were used for a targeted sequencing for each hypervariable segment.
(XLSX)

S2 Table Comprehensive statistical analysis between Cyprus and countries of interest. The genetic distances were calculated between Cypriots and countries of interest using the Arlequin software, providing information regarding their genetic differentiation.
(XLSX)

S3 Table Haplogroup frequencies among Cyprus and countries of interest, including references and sample sizes. Haplogroups from the current study and haplogroup data from previous studies regarding the countries of interest were combined and major haplogroup frequencies were calculated.
(XLSX)

S4 Table Haplogroup and subclades frequencies in Cypriots and 4 neighbouring countries. Haplogroup subclades found in Greek Cypriots, Armenian Cypriots, Maronite Cypriots, Greece, Lebanon, Syria and Turkey.
(XLSX)
